# Experience sharing of endoscopy-assisted total mastectomy with and without immediate breast reconstruction

**DOI:** 10.3389/fonc.2025.1711540

**Published:** 2025-11-05

**Authors:** Yidan Lin, Minxue Zhuang, Hongbin Qiu, Wei Chen, Yihui He, Huanhong Zeng, Mengbo Lin, Hui Zhang

**Affiliations:** 1 Department of Breast Surgery, Fuzhou University Affiliated Provincial Hospital, Fuzhou, Fujian, China; 2 Fujian Shengli Clinical Medical College of Fujian Medical University, Fuzhou, Fujian, China; 3 Department of Breast Surgery, Clinical Oncology School of Fujian Medical University, Fujian Cancer Hospital, Fuzhou, Fujian, China; 4 College of Integrative Medicine, Fujian University of Traditional Chinese Medicine, Fuzhou, Fujian, China; 5 Department of Pathology, Fuzhou University Affiliated Provincial Hospital, Fuzhou, Fujian, China

**Keywords:** breast cancer, endoscopy-assisted, axillary approach, total mastectomy, breast reconstruction

## Abstract

**Objective:**

To evaluate safety, efficacy, and aesthetics of endoscopy-assisted total mastectomy endoscope(EATM) with immediate breast reconstruction (IBR).

**Methods:**

This retrospective study analyzed 213 patients undergoing total mastectomy (2020.12–2023.2), stratified into four groups: conventional total mastectomy (CTM, n=128), EATM (n=46), CTM+IBR (n=17), and EATM+IBR (n=16). Operative metrics and patient-reported outcomes (Breast-Q/Scar-Q) were compared (SPSS 26.0, P<0.05).

**Results:**

EATM groups exhibited prolonged operative time compared to CTM (P < 0.05) but demonstrated significant advantages in reduced intraoperative bleeding (median: 50 mL vs. 80 mL), shorter incision length (3.2 cm vs. 8.5 cm), earlier drain removal (5 vs. 8 days), and shorter hospitalization (4 vs. 7 days) (P < 0.05). EATM with IBR (Group D) achieved superior breast tissue preservation and higher patient satisfaction in psychosocial health (Breast-Q score: 78 vs. 65) and scar appearance (Scar-Q score: 8.5 vs. 6.2) compared to conventional approaches (P < 0.05). Complication rates were comparable across groups (9.3% vs. 8.7%, P > 0.05), with only one case of local nipple recurrence and three cases of distant metastasis observed during 27-month follow-up.

**Conclusion:**

EATM combined with IBR represents a safe and effective strategy for breast cancer management, balancing oncological safety with enhanced aesthetic outcomes. The technique reduces surgical trauma, accelerates recovery, and improves patient satisfaction, particularly in scar concealment. Despite higher costs and procedural complexity, it is recommended for patients prioritizing both curative and cosmetic goals. Further multicenter studies are warranted to validate long-term efficacy and cost-effectiveness.

## Introduction

Breast cancer remains the most prevalent malignancy affecting women globally, with rising emphasis on balancing oncologic efficacy and quality-of-life outcomes (1). Advances in surgical techniques have transitioned from extensive resections to minimally invasive, aesthetically driven approaches, reflecting the paradigm shift toward personalized cancer care (2). Implant-based breast reconstruction has gained traction due to its technical feasibility and immediate cosmetic benefits. Current IBR strategies include direct-to-implant (DTI) and two-stage expander-implant (TE-I) techniques (3–8). Traditional approaches, however, often necessitate visible incisions (e.g., inframammary fold, periareolar), resulting in suboptimal scar concealment.

Endoscopic surgery, renowned for its minimally invasive nature and enhanced visualization, has revolutionized procedures in gynecology and general surgery (9–13). Nonetheless, endoscope applications in breast surgery have been limited by anatomical challenges, including restricted operative space and difficulties in pneumoperitoneum maintenance. As a result, the application of endoscope-assisted breast surgery has been limited. It was not until 1992 that Kompatscher first introduced endoscopic techniques into breast surgery (14). Advancements in single-port laparoscopy have enabled axillary access for radical mastectomy, combining concealed incisions with stable workspace establishment (15–18). The integration of single-incision axillary endoscopy-assisted total mastectomy endoscope (EATM) with immediate breast reconstruction (IBR) represents a transformative approach. This technique minimizes visible scarring, reduces postoperative morbidity, and aligns with patient demands for aesthetic preservation (19–22). Besides, this approach meets the desire of breast cancer patients for scar-free skin surfaces and perfect breast remodeling. It helps avoid the psychological and physical impacts caused by breast loss, contributing to reducing their psychological stress and improving overall well-being (2, 7, 23). Despite its potential, comparative data on EATM combined with IBR remain sparse.

This study evaluated the clinical outcomes and patient satisfaction of EATM with IBR, providing evidence to guide surgical decision-making in breast cancer management.

## Methods

### 2.1 Study design and patient selection

This retrospective cohort study reviewed 213 breast cancer patients who underwent radical mastectomy at Fuzhou University Affiliated Provincial Hospital (December 2020–February 2023). Participants were stratified into four groups based on surgical technique and reconstruction status:

1. Group A (*n* = 128): Conventional total mastectomy (CTM).
2. Group B (*n* = 46): Endoscopy-assisted total mastectomy endoscope (EATM).
3. Group C (*n* = 17): CTM with immediate breast reconstruction (IBR).
4. Group D (*n* = 16): EATM combined with IBR.

All patients provided written informed consent after comprehensive discussions of surgical risks and alternatives. All surgical procedures, encompassing the oncologic mastectomy and the immediate prosthetic reconstruction, were performed by the same surgeon who is dual-trained and highly experienced in both oncologic breast surgery and oncoplastic reconstruction techniques.

#### 2.1.1 Inclusion criterias

1. Females aged 21–41 years.
2. Unilateral breast cancer confirmed by histopathology.
3. Tumor characteristics: Ductal carcinoma *in situ* (DCIS) without size restriction; Invasive carcinoma ≤3 cm in diameter (post-neoadjuvant chemotherapy residual tumors ≤3 cm); Tumor distance >0.2 cm from gland surface, no chest wall fixation, skin dimpling, or peau d’orange changes.
4. Breast volume <500 mL, no significant ptosis (nipple position above inframammary fold).
5. Patients with a high aesthetic demand who, after comprehensive counseling on all oncologically sound options, explicitly chose mastectomy with immediate reconstruction for personal reasons.
6. No distant metastasis.

#### 2.1.2 Exclusion criterias

1. Bra size >E or post-mastectomy breast weight >600 g.
2. Nipple involvement or skin/pectoralis major muscle invasion.
3. Comorbidities: uncontrolled hypertension, diabetes, cardiac/renal/hepatic dysfunction.

### 2.2 Surgical method: EATM with IBR

#### 2.2.1 Special surgical equipment preparation

**Figure 1:**
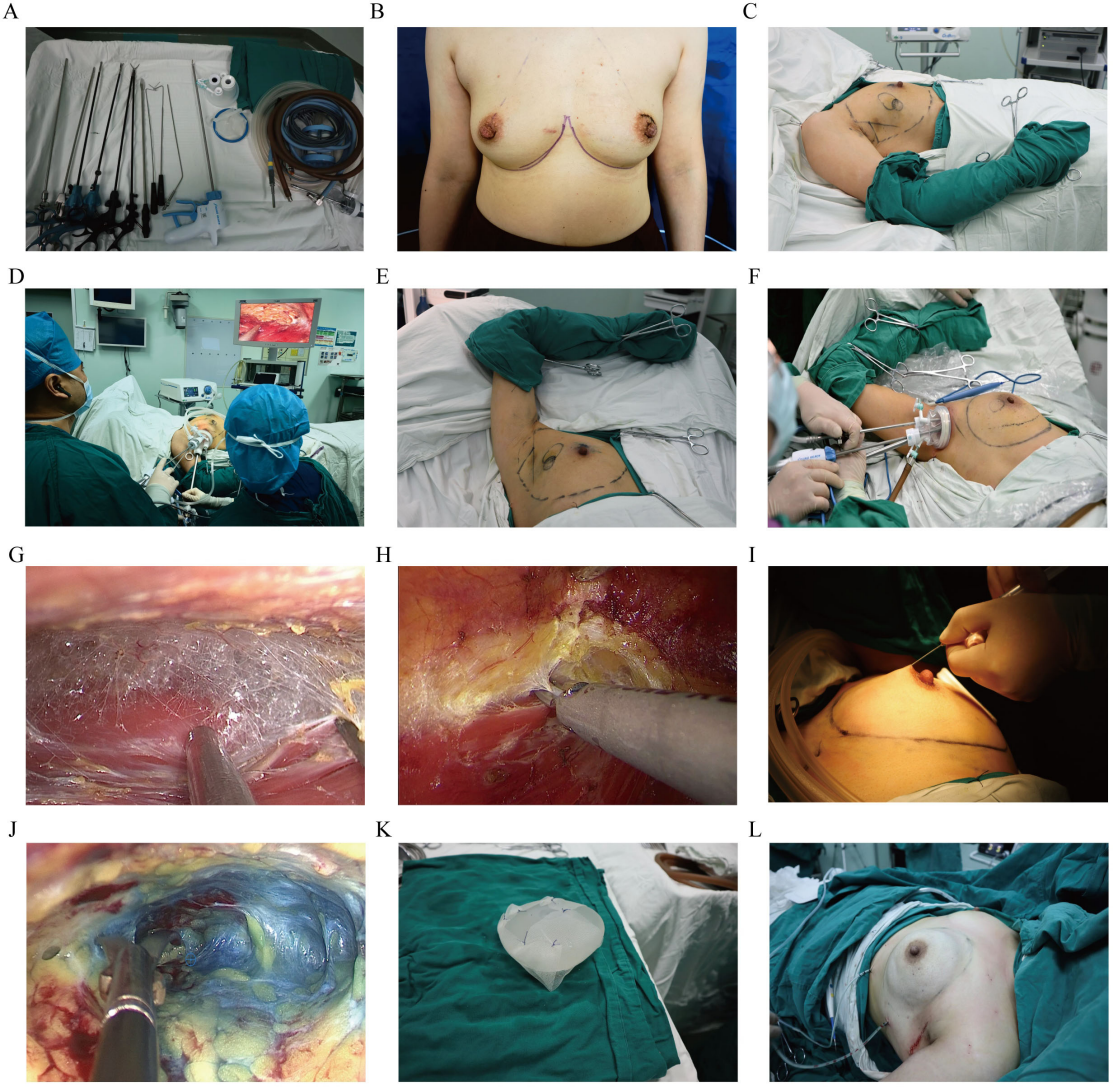
Surgical procedure of endoscopy-assisted total mastectomy, (EATM) combined with immediate breast reconstruction (IBR). **(A)** Specialized surgical instruments. **(B)** Preoperative markings of bilateral inframammary folds and mammary boundaries. **(C)** Patient positioning with ipsilateral thoracic elevation. **(D)** Surgeon and assistant roles during endoscope operation. **(E)** Affected arm fixation to optimize surgical space. **(F)** Incision protector and single-port endoscope device. **(G)** Ultrasonic scalpel dissection between pectoralis major and minor muscles. **(H)** Retro-mammary space dissection. **(I)** Subcutaneous adrenaline saline injection. **(J)** Retro-mammary space mobilization. **(K)** Mesh-wrapped prosthesis. **(L)** Post-reconstruction chest wall morphology.

A single-port endoscopic system (4-channel, 70 cm), long-handled ultrasonic scalpel, 30° laparoscope, Tiloop^®^ mesh (PFM Medical), and silicone implants were prepared (**Figure 1A**). All patients undergoing immediate breast reconstruction (Groups C and D) received a Tiloop^®^ Bra mesh (PFM Medical) to support the implant. The implants used were all round, smooth-shell silicone gel implants.

#### 2.2.2 Preoperative marking

**Figure 2:**
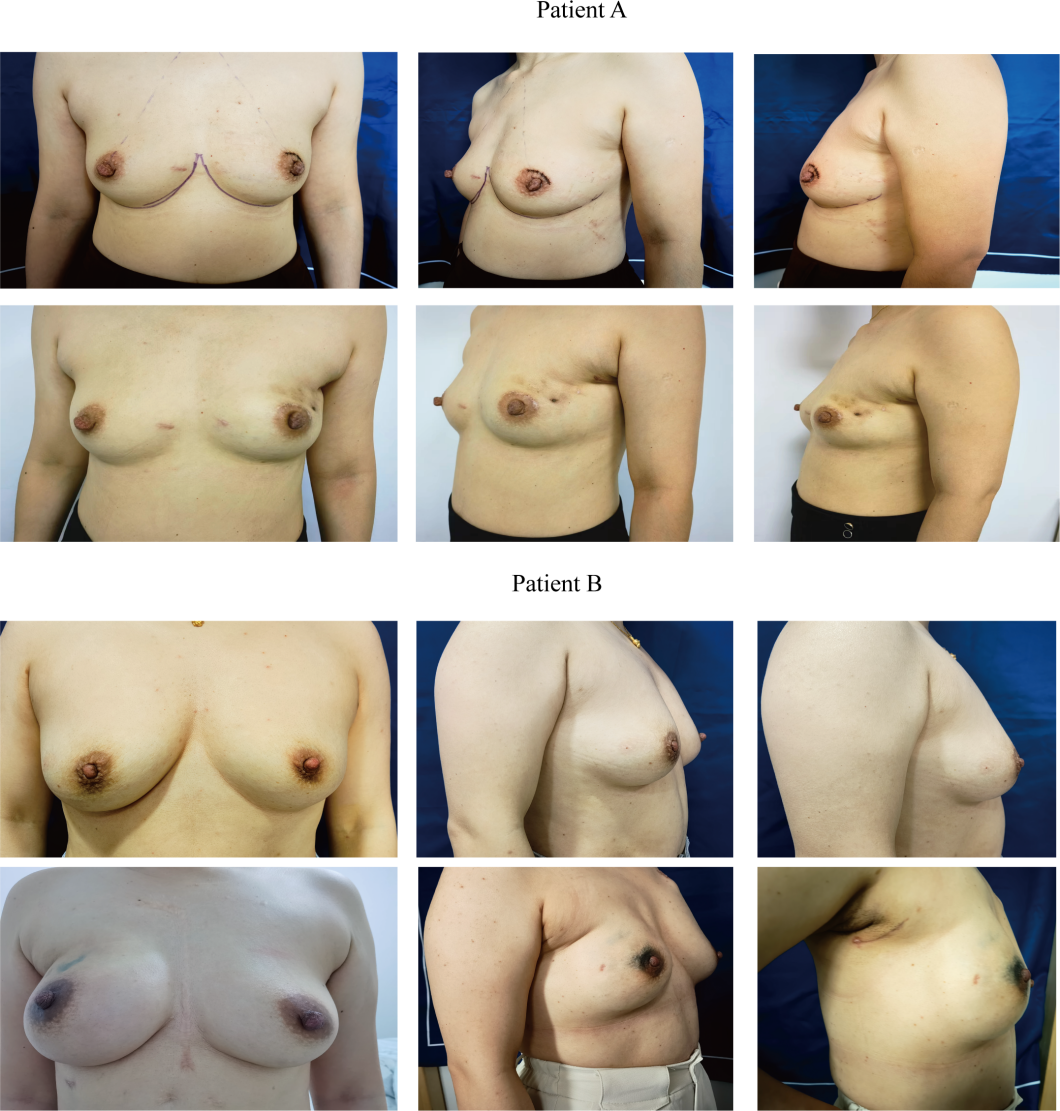
Preoperative and postoperative chest wall morphology in reconstruction group patients. Representative images at six months follow-up after EATM with IBR, demonstrating aesthetic outcomes and scar concealment.

Prior to surgery, the patient assumed an upright standing position for anatomical reference. The following anatomical landmarks were demarcated: (1) bilateral lower inframammary folds, (2) superior/medial/lateral mammary boundaries, and (3) A 3–4 cm curvilinear incision positioned horizontally within the axillary region. This incision was strategically placed at the midpoint between the anterior axillary fold and the breast margin, ensuring complete concealment during neutral arm positioning (**Figure 2B**).

For sentinel lymph node mapping, a dual-tracer protocol was implemented: 0.5 mL methylene blue (1% w/v) combined with 0.5 mL nano-carbon suspension (25 mg/mL) was administered via intradermal injection periareolarly (within the 3–9 o’clock quadrant) to optimize lymphatic visualization.

#### 2..32 Patient positioning

Following induction of general anesthesia, the patient was positioned in a standard supine orientation with the torso strategically aligned with the ipsilateral bed edge to optimize surgical access. The affected limb was configured as follows: (1) shoulder joint abducted to 90° with external rotation, (2) ipsilateral thoracic elevation to 30° using adjustable foam bolsters (**Figure 1C**). Routine draping and disinfection were performed, and the affected arm was wrapped with a sterile towel and fixed with towel clamps. The affected arm was then immobilized to the head (**Figure 1E**).

#### 2.2.4 Axillary approach

The initial 3–5 cm incision was strategically placed at the midpoint between the anterior axillary fold and the breast edge. In cases where ALND was anticipated or required, the incision could be extended posteriorly as needed to optimize exposure., ensuring it did not extend beyond the anterior axillary line. In cases of larger breast volume, the incision was extended posteriorly. Sentinel lymph node biopsy was performed under direct visualization. The stained or significantly enlarged axillary sentinel lymph node was identified and excised for intraoperative frozen section pathological examination. If the sentinel lymph node was found to be positive, axillary lymph node dissection was carried out following total mastectomy. The affected arm was flexed and secured to the head support, with the arm support then removed.

#### 2.2.5 Total mastectomy

Under direct visualization, the lateral edge of the pectoralis major muscle was exposed through the axillary incision, extending into the space between the pectoralis major and minor muscles. A wound protector was inserted, and a single-port endoscope device was connected (**Figure 1F**). Pneumoperitoneum was established using CO2 at a pressure of 8–10 mmHg (1 mmHg = 0.133 kPa) and a flow rate >20 L/min to create a working space for the mastectomy. Using an ultrasound knife, the loose connective tissue between the pectoralis major and minor muscles was separated (**Figure 1G**). The pectoralis major was transected up to the preoperative marking, ensuring the perforating blood vessels were preserved to avoid hemostasis complications, which could necessitate conversion to open surgery.

The wound protector was removed, and the space between the breast and the posterior tissue was cleared by approximately 2 cm. The wound protector and single-port endoscope device were reinserted, the pneumoperitoneum was reopened, and the working space was re-established. Using the ultrasound knife, the space behind the breast was freed (**Figure 1H**), including the pectoralis major fascia. This dissection extended from below the clavicle medially to the sternal margin, inferiorly to the inframammary fold, and laterally to the outer edge of the breast gland.

The endoscope device was removed, and adrenaline saline (1:500,000) was injected subcutaneously on the surface of the breast (**Figure 1I**). The anterior space of the breast was then dissected by approximately 2 cm under direct visualization. The wound protector and single-port endoscope device were reconnected, and the pneumoperitoneum was re-established. Using a dissector, the breast gland was retracted inferiorly to expose the superficial layer of the superficial fascia. Endoscope scissors were used for sharp dissection of the skin flap (**Figure 1J**), following the sequence: upper outer, posterior to the nipple, lower inner, upper inner, and lower outer. The anterior space of the breast was completely separated.

To achieve hemostasis, the endoscope scissors were then connected to an electrocautery hook. This helped prevent thermal damage to the skin flap caused by the ultrasound knife, which could lead to ischemia and necrosis of the nipple-areola complex (NAC). The gland was transected along the edge of the breast using an ultrasound knife. Tissue from behind the nipple was sent for intraoperative frozen section pathology. The procedure performed was a nipple-sparing mastectomy via an axillary approach, which involves the removal of all breast glandular tissue while preserving the NAC and the skin envelope.

The wound protector and single-port endoscope device were removed, and the specimen was extracted. Thorough lavage and hemostasis were performed. If the sentinel lymph node was positive, an axillary lymph node dissection was carried out. The pectoralis major muscle was retracted, and the fatty lymphatic tissue in the intermuscular space between the pectoralis major and minor was removed. The pectoralis minor muscle was retracted, and the clavicopectoral fascia was dissected to expose the axillary vein. The axillary lymphatic adipose tissue was carefully dissected along the axillary vein, ensuring precise protection of the long thoracic nerve, thoracodorsal nerve, and associated vessels. The lymphatic adipose tissue from axillary levels I and II was cleared. The ultrasound knife was used for precise hemostasis and sealing of lymphatic vessels, and the wound was irrigated with sterile injectable water.

#### 2.2.6 Breast reconstruction

Immediate breast reconstruction was performed following EATM. Cefazolin sodium (2.0 g) was administered 30 minutes prior to surgery as prophylaxis. After completing the mastectomy, the surgeon changed gloves and disinfected the skin. The volume of the excised breast was measured using the drainage method, and the diameter of the prosthesis was measured to determine the base width, ensuring proper prosthesis selection. The patch and prosthesis were soaked in a cefazolin sodium saline solution (2 g in 500 mL) for 5 minutes. A 2–0 microjo suture was used to secure the patch around the prosthesis (**Figure 1K**). The prosthesis and patch were placed in the subpectoralis muscle on the affected side. The lateral patch was sutured to the serratus anterior muscle to prevent lateral displacement of the prosthesis. The shape of both breasts was adjusted to achieve basic symmetry (**Figure 1L**).

A chest wall drain and an axillary drain were placed on the medial and lateral chest walls of the implant and fixed with continuous vacuum suction. The incisions were precisely sutured. A dressing was applied to the incision, and a compression bandage was placed on the upper edge of the prosthesis and armpit to maintain the shape. If the procedure lasted longer than 3 hours, additional cefazolin sodium (2.0 g) was given to prevent infection.

### 2.3 Postoperative care

Patients in the breast reconstruction group received 2.0 g of cefazolin sodium, administered twice daily (*bid*), for infection prophylaxis for two days after surgery. The patency of the chest wall and axillary drain was maintained with continuous negative pressure suction. The amount, color, and nature of the drainage fluid from the chest wall drain and axillary drain were observed and recorded daily. If the drainage volume was <20 mL over a 24-hour period for three consecutive days, the drainage tubes were removed.

When changing the dressing, careful attention was paid to the blood circulation of the NAC and the healing status of the incision to prevent ischemia and necrosis of the NAC as well as incision infection. Postoperatively, patients were advised to minimize limb activity and gradually strengthen the function of the affected upper limb over the course of two weeks. A post-mastectomy compression garment was worn for up to three months to help maintain the shape of the reconstructed breast. Subsequent treatment plans were determined based on the results of postoperative pathological and immunohistochemical examinations (24).

### 2.4 Observational parameters and criteria

The relevant outcome measures defined by these parameters would comprehensively assess the surgical outcomes, patient satisfaction, and recovery trajectory associated with the various surgical methods used in this study.

#### 2.4.1 Surgical-related parameters

Comprehensive patient data were extracted from the hospital’s electronic medical records. The data included duration of surgery, intraoperative bleeding, surgical incision length, duration with chest wall drain, duration with axillary drain, days in hospital, surgical cost, reconstruction time, and breast weight for both groups. Duration of surgery was defined as the total duration from skin incision to the completion of wound dressing. Days in hospital were defined as the time from the day of surgery to the day of discharge.

#### 2.4.2 Patient satisfaction

The Breast-Q 2.0, a validated tool, was used to assess patient-reported aesthetic outcomes. Follow-up was conducted via in-hospital questionnaires or phone interviews at 1 month and 3 months post-surgery to collect information on postoperative recovery, complications, and breast condition. The Breast-Q 2.0 evaluated patient satisfaction with their breasts at three time points: preoperative, 1 month postoperative, and 3 months postoperative.

Additionally, the tool evaluated satisfaction in other areas, including sexual life, psychosocial well-being, chest physical health, and abdominal physical health. The Scar-Q scale was also utilized at 1 month and 3 months postoperative to assess patient satisfaction with the appearance of scars.

Timing: The Breast-Q and Scar-Q questionnaires were administered at three specific time points: preoperatively, 1 month postoperatively, and 3 months postoperatively. Breast-Q Domains: We specified that the study evaluated the following core domains from the Breast-Q Reconstruction module: Psychosocial Well-being; Sexual Well-being; Physical Well-being (Chest); Satisfaction with Breasts; Physical Well-being (Abdomen). Scar-Q Domain: The Scar-Q was used specifically to assess satisfaction with scar appearance.

#### 2.4.3 Statistical methods

Data were analyzed using SPSS 26.0. Continuous variables were expressed as mean ± SD or median (IQR) and compared via *t*-test or Mann-Whitney *U* test. Categorical variables were analyzed by χ² or Fisher’s exact test. The significance threshold was set at *P* < 0.05.

#### 2.5 Ethical approval

This study strictly adhered to medical ethical standards and was approved by the Ethics Committee of Fuzhou University Affiliated Provincial Hospital. The research was conducted in full accordance with the principles outlined in the Declaration of Helsinki (2013 revision). Written informed consent was obtained from all enrolled patients or their authorized family members.

## 3 Results

### 3.1 Baseline characteristics

The study enrolled 213 patients, categorized into radical surgery cohorts (Groups A/B: *n* = 174) and reconstruction cohorts (Groups C/D: *n* = 39). Demographic and clinical characteristics demonstrated comparability across all groups (**Table 1**).

**Table 1:** Baseline demographic and clinical characteristics of patients stratified into four groups.

Characteristics	Group A(n=132)	Group B(n=48)	P value	Group C(n=17)	Group D(n=18)	P value
Mean age , years	36(33-38)	37(35-40)	0.076	37(35-40.5)	36.5(32-38.8)	0.191
Mean BMI (SD), kg/m^2^	22.05 (20.44-23.44)	21.07 (20.10-23.58)	0.321	21.80 (20.5-22.53)	20.18 (18.80-21.88)	0.081
Smoking status						
Yes	11 (6.1%)	2 (1.1%)	0.529	17 (48.6%)	17 (48.6%)	1.000
No	121 (67.2%)	46 (25.6%)		0 (0%)	1 (2.9%)	
Background diseases						
Yes	41 (22.8%)	12 (6.7%)	0.430	2 (5.7%)	1 (2.9%)	0.603
No	91 (50.6%)	36 (20%)		15 (42.9%)	17 (48.6%)	
Neoadjuvant, N (%)						
Yes	47(35.6)	12(25.0)	0.180	4(23.5)	4(25.0)	0.619
No	85(64.4)	36(75.0)		13(76.5)	12(75.0)	
Pathological type						
Invasive carcinoma	122(92.4)	40(83.3)	0.129	15(88.2)	12(75.0)	0.298
Non-invasive cancer	10(7.6)	8(16.7)		2(11.8)	4(25.0)	
Subtype						
Luminal A	69(52.3)	24(50.0)	0.976	13(76.5)	13(81.3)	1.000
Luminal B	24(18.2)	10(20.8)		2(11.8)	2(12.5)	
HER-2+	21(15.9)	7(14.6)		1(5.9)	1(6.3)	
TNBC	18(13.6)	7(14.6)		1(5.9)	0(0.0)	
Tumer size, cm						
≤2	77(58.3)	26(54.2)	0.790	12(70.2)	10(62.5)	0.581
>2,≤5	53(40.2)	22(45.8)		4(23.5)	6(37.5)	
>5	2(1.5)	0(0.0)		1(5.9)	0(0.0)	
Location, N (%)						
Upper outer quadrant	76(57.6)	27(56.3)	0.435	13(76.5)	10(62.5)	0.067
Outer lower quadrant	17(12.9)	11(15.6)		0(0.0)	4(25.0)	
Upper inner quadrant	29(22.0)	9(18.8)		1(5.9)	2(12.5)	
Inner lower quadrant	6(4.5)	1(2.1)		2(11.8)	0(0.0)	
Multiple tumors	4(3.0)	0(0.0)		1(5.9)	0(0.0)	
Axillary surgery, N (%)						
SLNB+ALND	64(48.5)	16(33.3)	0.070	6(35.3)	5(31.3)	0.549
SLNB+SLNB	68(51.5)	32(66.7)		11(64.7)	11(68.8)	
Nipple posterior tissue(%)						
Negative	–	–	–	16(94.1)	16(100.0)	0.515
Positive	–	–		1(5.9)	0(0.0)	
Size of implants, mean ± sd	–	–	–	280.88 ± 28.297	266.39 ± 43.515	0.254
Clinical AJCC stage						
I	70(53.0)	23(47.9)	0.514	10(58.8)	7(43.8)	1.000
II	47(35.6)	22(45.8)		5(29.4)	9(56.3)	
III	14(10.6)	3(6.3)		2(11.8)	0(0.0)	
IV	1(0.8)	0(0.0)		0(0.0)	0(0.0)	

Group A, (Conventional total mastectomy, CTM); Group B, (Endoscopy-assisted total mastectomy, EATM); Group C, (Conventional total mastectomy with immediate breast reconstruction, CTM with IBR); and Group D, (Endoscopy-assisted total mastectomy endoscope with immediate breast reconstruction, EATM with IBR).

#### 3.1.1 Group A & group B

Group A comprised 132 patients, with an age range of 28–58 years, a median age of 36 years (IQR: 33–38), and a BMI of 22.05 (IQR: 20.44–23.44). Group B consisted of 48 patients, with an age range of 27–41 years, a median age of 37 years (IQR: 35–40), and a BMI of 21.07 (IQR: 20.10–23.58). No statistically significant intergroup differences were observed in age, BMI, smoking status, background diseases, marital status, preoperative chemotherapy, tumor size, pathological type, molecular subtype, or tumor stage (*P* > 0.05).

#### 3.1.2 Group C & group D

Group C included 17 patients, with an age range of 28–54 years and a median age of 37 years (IQR: 35–40.5). Group D comprised 18 patients, with an age range of 23–41 years and a median age of 36.5 years (IQR: 32-38.8). The two groups exhibited no significant differences in age, BMI, smoking status, background diseases, marital status, preoperative chemotherapy, pathological type, molecular subtype, tumor size, size of implants or tumor stage (*P* > 0.05), confirming baseline comparability. **Figure 2** illustrates the chest wall morphology of two representative patients at six months postoperatively following EATM with IBR.

### 3.2 Surgical outcomes

All procedures were completed successfully, with no cases requiring conversion to open surgery in Groups B and D.

#### 3.2.1 Comparative analysis of surgical metrics in groups A & B

**Figure 3:**
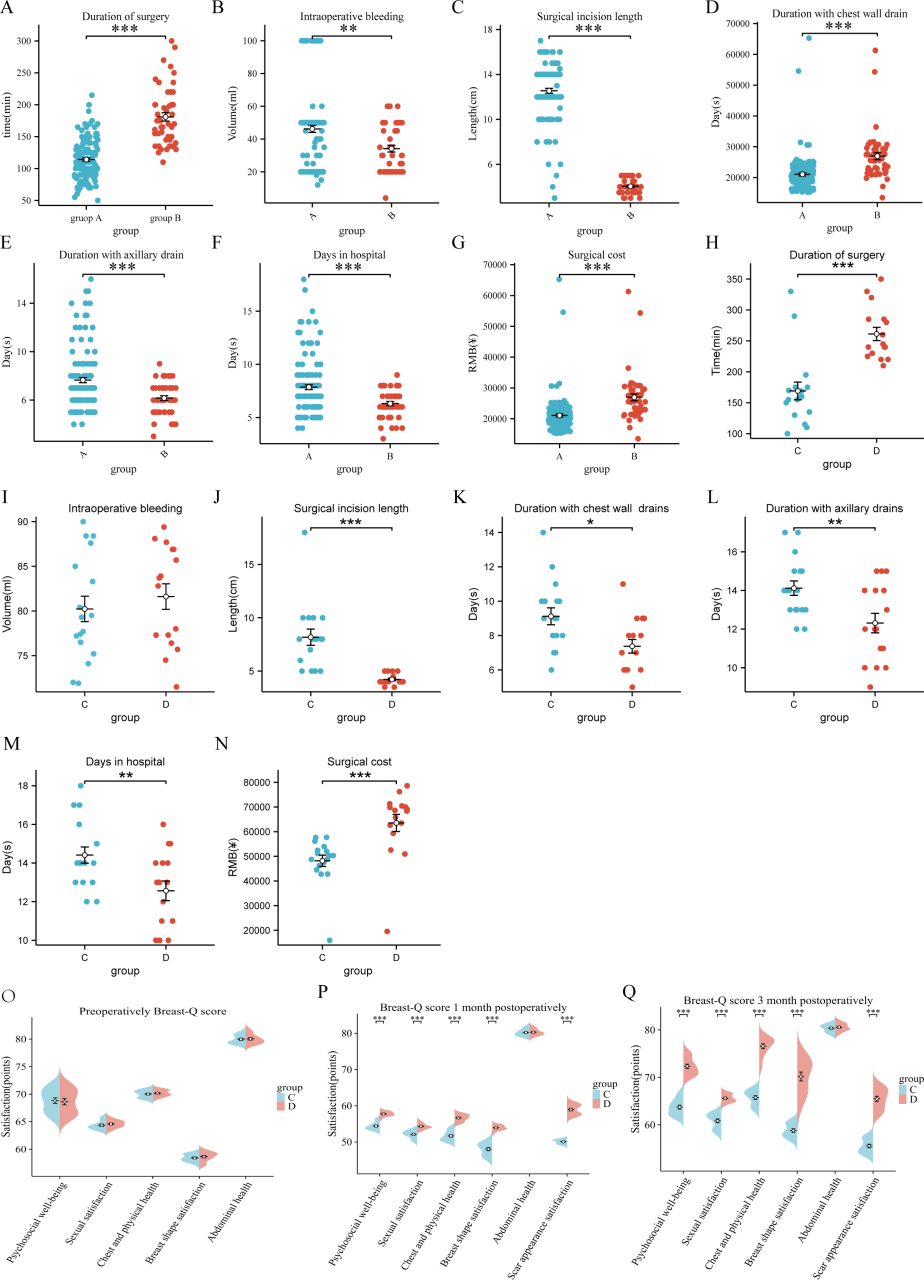
Comparative analysis of surgical and patient-reported outcomes. **(A-G)** Radical surgery cohort (Group A vs. Group B): Operative time, intraoperative blood loss, incision length, chest wall/axillary drain duration, hospitalization days, and surgical costs. **(H-N)** Reconstruction cohort (Group C vs. Group D): Operative time, intraoperative blood loss, incision length, drain duration, hospitalization days, and costs. **(O-Q)** Breast-Q scale scores (preoperative, 1-month, and 3-month postoperative) for psychosocial health, sexual satisfaction, chest physical health, breast appearance, and abdominal well-being. Significance levels: *P < 0.05, **P < 0.01, ***P < 0.001.

Patients in Group A exhibited a longer duration of surgery compared to Group B (**Figure 3A**, P < 0.05). However, Group A demonstrated superior outcomes in terms of intraoperative bleeding, surgical incision length, duration with chest wall drain, duration with axillary drain, and days in hospital (**Figures 3B–F**, P < 0.05).

#### 3.2.2 Comparative analysis of surgical metrics in groups C & D

The duration of surgery was prolonged in Group D relative to Group C (**Figure 3H**, P < 0.05). Notably, Group D achieved favorable results in breast tissue mass preservation, minimized incision length, expedited drain removal, and reduced postoperative hospital stay (**Figures 3J–M**, P < 0.05). No statistically significant differences were observed between the groups in reconstruction duration or blood loss (**Figure 3I**, P > 0.05).

### 3.3 Postoperative complications

#### 3.3.1 Group A & group B

Group A experienced 9 postoperative adverse events: 3 cases of postoperative hemorrhage, 3 cases of subcutaneous seroma following drain removal, 1 case of surgical site infection, and 2 cases of upper limb thrombosis. In contrast, Group B reported 4 complications: 2 instances of postoperative hemorrhage, 1 case of limb thrombosis, and 1 case of subcutaneous emphysema. No surgical site infections or seromas were documented in Group B.

#### 3.3.2 Group C & group D

In Group C, a single case of ascending infection occurred secondary to prolonged drain retention. Group D exhibited 3 complications: 1 case of subcutaneous emphysema and 2 cases of prosthetic-related infection. The latter included one infection attributed to ascending pathogens and another due to inadvertent drain dislodgement. Both prosthetic infections were resolved with targeted intravenous antibiotics and surgical irrigation, enabling preservation of the breast implant.

### 3.4 Patient-reported outcomes and complications in group C and group D

Preoperative Breast-Q scores for psychosocial well-being, sexual satisfaction, chest physical well-being, breast appearance satisfaction, and abdominal physical well-being demonstrated no significant intergroup differences between cohorts C and D (**Figure 3O**, P > 0.05). At the 1-month postoperative follow-up, both groups exhibited reduced Breast-Q scores compared to baseline. However, Group D achieved significantly higher scores than Group C in psychosocial well-being, sexual satisfaction, chest physical well-being, and breast appearance satisfaction (**Figure 3P**, P < 0.05). By 3 months postoperatively, scores for all domains (psychosocial well-being, sexual satisfaction, chest physical well-being, and breast appearance satisfaction) surpassed both preoperative and 1-month postoperative levels in both groups. Group D maintained statistically superior satisfaction scores relative to Group C (*P* < 0.05), whereas abdominal physical well-being satisfaction remained comparable between groups (**Figure 3Q**, P > 0.05). Scar-Q assessments revealed significantly higher scar appearance satisfaction in Group D compared to Group C at both 1-month and 3-month postoperative evaluations (*P* < 0.05).

### 3.5 Cost analysis

In the radical surgery cohort, the mean total cost for Group A was 20,344.5 yuan (range: 17,994.5–23,024.3 yuan), whereas Group B incurred a higher mean cost of 25,901.5 yuan (range: 25,354.7–31,482.7 yuan). Within the reconstruction cohort, Group C demonstrated a mean cost of 53,221 yuan (range: 50,284–57,641 yuan), compared to 71,088 yuan (range: 68,483–76,936 yuan) for Group D. Endoscopy-assisted procedures were associated with significantly elevated costs relative to conventional surgical approaches in both cohorts (**Figures 3G, N**, P < 0.05), imposing a greater financial burden on patients. This cost disparity represented a critical barrier to the widespread adoption of single-incision endoscope-assisted techniques.

### 3.6 Oncological safety

All patients underwent rigorous follow-up for 27 months. No significant intergroup differences were observed in patient-reported satisfaction scores for bilateral breast symmetry or NAC sensory preservation. To date, the study documented one case of local nipple recurrence and three cases of distant metastasis across both radical and reconstruction groups. Surgical protocols strictly adhered to nipple-sparing mastectomy guidelines, with intraoperative frozen-section analysis revealing positive subareolar margins in three cases, necessitating concurrent NAC excision. These findings suggested that EATM achieved recurrence and survival rates comparable to traditional open surgery, affirming their oncological safety.

## 4 Discussion

Breast cancer remains the most prevalent malignancy among women globally, with rising incidence rates in China (1). Surgical intervention is pivotal in comprehensive breast cancer management. The evolution of minimally invasive and oncoplastic techniques has expanded therapeutic options, prioritizing both oncologic safety and quality-of-life outcomes (2, 25). Conventional approaches often leave visible breast scars, which may negatively impact patients’ psychosocial well-being (26). In contrast, EATM combined with IBR offers concealed incisions and improved aesthetic outcomes without compromising oncologic efficacy (7, 8, 27). The skin tissue of the breast does not contribute to the local recurrence of breast cancer. The residual ductal epithelium of the breast is the primary cause of local recurrence of breast cancer (28), preservation of the skin and nipple-areolar complex does not increase the rate of local recurrence or distant metastasis (25). The use of the reverse order method for tissue separation better adheres to the tumor-free principle, significantly reducing muscle and tissue tension and compression, while effectively improving postoperative aesthetics and patient satisfaction (7). With the continuous accumulation of practical experience in EATM with IBR, an increasing number of detailed issues need to be reconsidered and objectively evaluated in order to fully leverage and utilize the advantages of this technique.

In this study, we reviewed 213 breast cancer patients who underwent CTM, (Group A), EATM (Group B), CTM with IBR (Group C), and EATM with IBR (Group D). Our findings elucidated the advantages and considerations associated with each method. The results showed that EATM (with or without IBR) required longer operative time compared to CTM. This delay was attributed to patient positioning, endoscope equipment setup, and the learning curve for surgeons during their initial phase of adopting the technique. As surgical experience accumulated, the learning curve for EATM shortened, approaching the operative time of traditional mastectomy. Patients undergoing EATM (with or without IBR) demonstrated significantly reduced intraoperative bleeding, shorter incision length, earlier drain removal, shorter hospital stay, and decreased duration with chest wall and axillary drains (P < 0.05). The rates of seroma and infection observed in our cohort, particularly in the EATM groups, were lower than those reported in some larger series. We postulate that this may be attributable to the synergistic effect of meticulous endoscopic hemostasis, a standardized and conservative drain management protocol, and the selection of a lower-risk patient population without major comorbidities. The adoption of the endoscopic technique incurred a significant additional cost of approximately 18,000 CNY per case when combined with IBR, primarily attributable to the use of specialized single-port equipment. This financial disparity represents a critical barrier to its widespread adoption and must be weighed against the demonstrated benefits in aesthetic outcome and patient satisfaction.

The axillary single-port endoscope approach shifts the surgical incision from the anterior chest to the axilla, avoiding tension on the breast surface that could lead to scar widening or incision rupture. This technique also alleviates implant-related tension on the incision, reducing the risk of poor wound healing, implant exposure, or loss (29, 30). The single axillary incision proved to be a versatile and adequate access point not only for the mastectomy and reconstruction but also for a formally oncologic ALND when required, thereby avoiding additional scars on the breast or chest wall. The endoscope magnification (8–10×) and illumination enhance visualization of fascial and ligamentous structures, nerve pathways, and vasculature, thereby minimizing intraoperative bleeding and iatrogenic injury (17, 31–33). These advantages facilitated earlier drain removal and shorter hospitalization. Although complications such as nipple ischemia, subcutaneous emphysema, infection, thrombosis, and bleeding occurred in a minority of cases, their low incidence precluded definitive statistical conclusions. Both conventional and endoscope approaches ensured oncological safety. The oncological safety of preserving the NAC is predicated on rigorous patient selection and intraoperative confirmation of negative subareolar margins. Contrary to historical concerns, a growing body of evidence suggests that in appropriately selected patients—such as those in our cohort with tumors located more than 2 cm from the nipple and without skin involvement—NSM does not appear to confer a higher risk of local recurrence or compromise survival outcomes compared to other mastectomy techniques. It is the residual ductal epithelium in the breast parenchyma, rather than the skin or NAC perse, that is the primary source of local recurrence, provided the NAC is free of disease.

The BREAST-Q scale, developed by Pusic et al., is widely used to assess breast reconstruction outcomes across domains including psychosocial health, sexual satisfaction, chest and abdominal physical well-being, and aesthetic satisfaction (34). Our findings suggested that avoiding breast surface incisions and optimizing chest wall morphology improved patient satisfaction with breast appearance, body image perception, and psychological well-being. However, EATM with IBR has not yet become a mainstream approach due to procedural complexity, high implant costs, steep learning curves, and elevated surgical expenses.

However, the interpretation of our findings must consider several limitations. The retrospective, single-center design inherently carries a risk of selection bias, while the small and uneven cohort sizes limit the statistical power and generalizability of the conclusions. Furthermore, the median follow-up of 27 months precludes definitive assessment of long-term oncological safety and implant-related complications. Therefore, this study serves to demonstrate the feasibility of the technique and generate hypotheses, rather than to provide conclusive evidence.

## 5 Conclusion

In conclusion, EATM, whether combined with IBR or not, represents an innovative surgical strategy for breast cancer management. This approach demonstrated notable advantages in operative feasibility, oncological safety, and aesthetic outcomes (particularly scar concealment), while enhancing postoperative quality of life and patient satisfaction. Our findings support its clinical applicability as a balanced therapeutic option for patients prioritizing both curative and cosmetic goals.

## Data Availability

The raw data supporting the conclusions of this article will be made available by the authors upon reasonable request, without undue reservation, and in compliance with ethical standards for patient data.
