# Correction: Experience sharing of endoscopy-assisted total mastectomy with and without immediate breast reconstruction

**DOI:** 10.3389/fonc.2026.1771995

**Published:** 2026-01-30

**Authors:** Yidan Lin, Minxue Zhuang, Hongbin Qiu, Wei Chen, Yihui He, Huanhong Zeng, Mengbo Lin, Hui Zhang

**Affiliations:** 1 Department of Breast Surgery, Fuzhou University Affiliated Provincial Hospital, Fuzhou, Fujian, China; 2 Fujian Shengli Clinical Medical College of Fujian Medical University, Fuzhou, Fujian, China; 3 Department of Breast Surgery, Clinical Oncology School of Fujian Medical University, Fujian Cancer Hospital, Fuzhou, Fujian, China; 4 College of Integrative Medicine, Fujian University of Traditional Chinese Medicine, Fuzhou, Fujian, China; 5 Department of Pathology, Fuzhou University Affiliated Provincial Hospital, Fuzhou, Fujian, China

**Keywords:** breast cancer, endoscopy-assisted, axillary approach, total mastectomy, breast reconstruction

Affiliation 1 Department of Breast Surgery, Fuzhou University Affiliated Provincial Hospital, Fuzhou, Fujian, China was erroneously given as 2 Department of Breast Surgery, Fuzhou University Affiliated Provincial Hospital, Fuzhou, Fujian, China.

Affiliation 2 Fujian Shengli Clinical Medical College of Fujian Medical University, Fuzhou, Fujian, China was erroneously given as 3 Fujian Shengli Clinical Medical College of Fujian Medical University, Fuzhou, Fujian, China].

Affiliation 3 Department of Breast Surgery, Clinical Oncology School of Fujian Medical University, Fujian Cancer Hospital, Fuzhou, Fujian, China was erroneously given as 1 Department of Breast Surgery, Clinical Oncology School of Fujian Medical University, Fujian Cancer Hospital, Fuzhou, Fujian, China.

Affiliation 5 Department of Pathology, Fuzhou University Affiliated Provincial Hospital, Fuzhou, Fujian, China was erroneously given as 5 Department of Pathology, Fujian Provincial Hospital, Fuzhou, Fujian, China.

The affiliations of the authors in the author list of the published paper was erroneously given as:

Yidan Lin^1†^, Minxue Zhuang^2,3†^, Hongbin Qiu ^4†^, Wei Chen^2,3†^, Yihui He ^3,5^, Huanhong Zeng^2,3^, Mengbo Lin^2,3^* and Hui Zhang^2,3^*

The correct affiliations of the authors in the author list reads:

Yidan Lin^3†^, Minxue Zhuang^1,2†^, Hongbin Qiu^4†^, Wei Chen¹,²^†^, Yihui He²,^5^, Huanhong Zeng¹,², Mengbo Lin¹,²* and Hui Zhang¹,²*

The original version of this article has been updated.

